# Evaluation of biological measures and multipurpose adaptive grass on soil bund in Lasta district, Ethiopia

**DOI:** 10.1016/j.heliyon.2023.e18198

**Published:** 2023-07-12

**Authors:** Haymanot Lamesgn Zena, Yalelet Abie

**Affiliations:** Sekota Dryland Agricultural Research Center, P.O Box 62, Sekota, Ethiopia

**Keywords:** Biological measures, Green biomass, Land productivity, Moisture content, Soil bund, Survival rate

## Abstract

The adverse effect of soil erosion is a major problem in Ethiopia, and soil and water conservation efforts must do to reduce the impact. Use of biological measures (grasses) combined with soil bund have numerous impact for reducing soil nutrient loss and increase soil moisture conservation, secure animal fodder for farm owners in low grass potential areas, enhance productivity of land and green biomass, but its adoption has been limited in the study area. This study explored the effect of grasses combined with soil bund on the experiment want to see the effect of stabilizer grasses on soil bund have seven treatments with randomized complete block design. Moisture content and bulk density data were collected and analyzed, taken undisturbed soil sample by gravimetric method, survival rate available plant per total planted times 100, tiller total number, plant height via meter and biomass using hanging balance data's were collected. The data analysis was done using R-Software and for mean separation, LSD at 5% significance level was used for moisture, bulk density, survival rate, biological parameters. Grass have positive impact on moisture content and bulk density to increase ease of use of water for grass and to stabilize the bund results in 2020 was 22.2%, 17.56%, and 12.3% of difference vetiver, Sudan grass, elephant and panicum in 2021 13% (1.36) in Sudan grass with comparison of the control treatment (1.57), respectively. Sudan grass and panicum have (100%) and (80%) performance on survival rate to rehabilitate and support the bund and protect direct runoff. Panicum has scored 77.2 average tillers in 0.15 m^2^ area on number of tiller that can affect biomass and direct runoff. Sudan grass 98.7 cm, elephant 85.4 cm and panicum 81 cm was resulted in 2021 and in 2020 Sudan grass 136.4 cm, elephant 91 cm and panicum 78.3 cm record on plant height. The green biomass that have great contribution for forage and other multipurpose use was Sudan grass, elephant and panicum yielded 20.8 t/ha in 2020 12.7 t/ha and 10.6 t/ha in 2022 respectively. Overall, in the experiment Sudan grass, Panicum Coloratum and Elephant grass have better adaptability and survival, increase farm land productivity contributing additional grass proceeds and have multipurpose use of fodder production.

## 1 Introduction

Ethiopia is one of the most bio-diverse countries in the world, with 79% of the population working in agriculture. In contrary, one of the countries with increasing degradation of soil fertility and water quality, biodiversity loss, deforestation, and mainly by soil erosion [1].

Lack of adequate soil protection measures and poor land use management plays a major role in the country's severe soil erosion problem, with an average annual soil loss rate of 30.2 t ha^-^1 yr^-^1 recorded.

Those problems are product estimated minimum soil loss become 12.1 t/ha/yr around the Kogagawa estuary, which is larger than the minimum allowable soil loss (2 t/ha/yr) [2]. To tackle from Sevier erosion and soil loss by soil embankments of farmlands is one method, complemented by biological and agronomic measures help improve production, in order to improve adaptability to local conditions is needed [3].

The grass is one of the biological countermeasures among those Panicum coloratum grass one of provides excellent forage for livestock. It is commonly use as forage or hay for animals. The plant produces an abundance of high quality forage has many other conservation benefits including: soil stabilization and re-vegetation on depleted soils or range condition. It can also be used to prevent soil erosion on embankment, ditches, farm lands, and other highly erodible sites [4]. The species seem promising as forage species to be introduced in temperate, lowland areas prone to soil flooding [5].

Sudan grass is essential for the dry-steppe zone and most productive and drought-resistant, as well as promising culture [6].

Elephant grasses, an important tropical grass and one of the highest-yielding tropical grasses and a versatile species that can be grown in a wide diversity of conditions and systems. Nowadays, an increasing interest in producing feeds is imperative to achieving economic and sustainable goals dry or wet conditions, small or large scale farming [7]. It is a valuable fodder and very popular in the tropics, especially in cut-and-carry systems [8]. Panicum coloratum originates from Africa and is now found in many tropical and subtropical regions [9], between 30°N and 33°S, from sea level to an altitude of 2100 m [9]. Panicum coloratum grows best during the warm season, with temperatures ranging from 18 °C to 36 °C, with an annual mean temperature around 22 °C, and annual rainfall ranging from 400 to 2000 mm (depending on the variety), on fertile sandy to clay soils. Panicum coloratum is drought tolerant and moderately tolerant of flooding and waterlogged conditions. Var. makarikariense is particularly suitable for flooding conditions [10]. Panicum coloratum can withstand significant levels of salinity. It is susceptible to frost but can recover after it. It also recovers from fire [10].

Use soil and water conservation with biological measures are one of the most important practices for conserving soil and water structures, biodiversity and increase agricultural land productivity through soil conservation measures (grasslands, erosion ditches, dikes, hedges, and terraces). It's one refinement of research promotion of the feed market and feed research is desired [11]. The importance of bund stabilization with Desho grass and others is not well known until recent time [12]. And there is a loss of nutrient, soil and water from cultivated land in soil bund without supporting by biological measures as [13] conclude and the importance of stabilizers in the mandate area are not supported by research and the agro ecology of the study area are also not well addressed.

The aim of the experiment was to evaluate and adopt the Contribution of biological measures grasses that are not practiced in the study area, conservation structure stabilizer demonstration, availability of choice of different grass and increase stability of the soil with integration of soil and water conservation practices (SWC).

Forage availability for animals, to convince the perception of farmers on SWC practices is minimizing the farm size rather than minimizing soil erosion, mitigation of degraded rangelands, fodder and other ornamental importance. There for this study was be offered to evaluate different adaptive biological grass on soil bund and its multipurpose uses.

## 2 Material and method

### 2.1 Area description

**Fig. 1:**
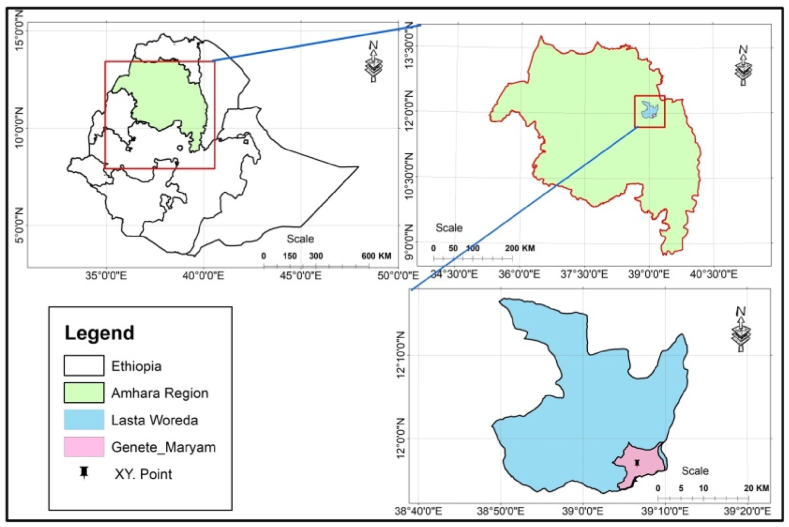
Map of study area.

The experiment conducted as Fig. 1 expresses were in North Wollo administrative zone the specific area of Genete Maryam 17 km from Lalibela and found in Lasta district. The location is 11°56^’^58.11″N latitude, 39^0^06′35.81″E longitude and the Elevation is 2326 m m.a.sl. Annual rainfall, maximum and minimum temperature in 2020 was 979.2 mm, 24.5 °C and 13.5 °C respectively and in 2021 was 1027.2 mm, 24.3^0^c and 13.6^0^c respectively. The trial field soil characteristics are 7.5 soil pH, 0.067% organic matter and 26.97 ppm available phosphors.

The trial site slope was recorded 12% gentle slope were soil bund is recommended in the area. The growing periods mostly from beginning of July until end of October. As the rainfall usually stops early, particularly at flowering stages of local grass of the area and major crops, the availability of low soil moisture content at this stage and low soil fertility status of most agricultural lands are the major limiting factors for most grass and shrubs production in the study areas.

The study was conducted in Lasta district for two years from 2020 to 2021. The study area was select by using purposive sampling method for stable trial establishment. The trial was done on soil bund with grass. Six grasses selected based on their adaptability and multipurpose use. The design for the experiment was arrange in randomized complete block design (RCBD) with three replications.

1 **Vetiver** (*Vetiverialzizanioides*) spacing of vetiver between rows are 1–2 m areas which is appropriate for soil bund and the space between plants are 50 cm [14],
2 **Elephant** (*Pennisetumpurpureum*) Elephant grass produces very few seeds and is mostly propagated vegetative through stem cuttings consisting of at least 3 nodes, 2 of which are buried in rows. Row width ranges from 50 to 200 cm and distance within rows is between 50 and 100 cm [9].
3 **Desho** (*Pennisetumpedicelluatum*) the spacing between rows to be 50 cm and between plants are 50 cm [15],
4 **Sudan grass** (Sorghum Sudanense) the spacing between rows is 25 cm and drilling [16],
5 **Rhodes** (*Chlorisgayana*)the spacing of Rhodes grass between row is 30 cm and root splitting its seed rate is 15 kg/ha [17] and will put on constructed soil bund found on each field. Each grass will plant on strip of soil bund with recommended planting pattern (Table 1). Grasses plant in 5 m length and the standard width of soil bund, 1 m interval between treatments

**Table 1:** Planting pattern of grass and bund size.

Grass stabilize/	Planting method	Space between row	Space between plant	Bund size in meter
*Vetiveriazizanioides*	Root split	1–2 m	0.5 m	3*(0.5–0.75 m)
*Pennisetumpurpureum*	Root split	0.5–2 m	o.5-1 m	3*(0.5–0.75 m)
*Pennisetumpedicellatum*	Root split	0.5 m	0.5 m	3*(0.5–0.75 m)
*Sorghum sudanens*	Seed dressing	0.5 m	0.5 m	3*(0.5–0.75 m)
*Chlorisgayana*	Root split	0.5 m	0.5 m	3*(0.5–0.75 m)
*Panicum coloratum*	Root split	0.5 m	0.5 m	3*(0.5–0.75 m)
6. **Panicum** (panicum coloratum) the spacing of panicum grass between row is 30 cm and root splitting its seed rate is 10 kg/ha

#### 2.1.1 Treatment design

1. Soil bund with Desho grass (*P. pedicelluatum*) (*SB + Dg*)
2. Soil bund with Elephant grass (*Pnnisetumpurpureum*) (*SB + Eg*)
3. Soil bund with *Vetiver* (*V. zizanioides*) (*SB + Vs*)
4. Soil bund with Sudan grass (SB + SG)
5. Soil bund with Rhodes (*Chlorisgayana) (SB + RO) and*
6. Panicum coloratum (SB + PC)
7. Soil bund only (SB)

#### 2.1.2 Soil bund design and construction

Soil bunds in the study area construct based on the soil and water conservation guideline of the Ministry of Agriculture [18] uses for control erosion, increases soil moisture, reduce slop length their by improve land productivity and there will be maintenance of soil bunds to make appropriate for the trial. The horizontal distance between two successive soil bunds determine based on the vertical interval between bunds (usually 1 m for Ethiopia) and the slope angle [18]. The base width and top width of the bund (embankment) from 1 m to 1.2 m and 0.30 m to 0.50 m respectively, the channel 0.3 m deep and 15 cm berm will have. Besides, the height of the bund will 0.60 m after compaction as described in Table 1.

### 2.2 Data collection

#### 2.2.1 Agronomic data

Data on biological performance of grass, Morphological parameters as such plant height and tillers was measured from five (5) plants randomly selected from rows of each soil bund after planting then compute as mean counts. To determine biomass yield, the forage frequency of harvest done by hand using a leaving sickle a stubble height of 8 cm according to recommended practice. The fresh herbage yield measured immediately after each harvest using a portable balance with a sensitivity of 0.01 g. Survival data of the adaptable multipurpose grass was done by available plant count per total planted plant of the grass.

#### 2.2.2 Soil data

A soil composite sample collected from 0 to 20 cm on representative points in the trial sites to examine in the laboratory for major physiochemical properties and soil moisture characteristics. The USDA textural classification triangle was used to define the textural class for each composite soil samples taken [19].

Besides, additional soil samples was taken from each treatment every 2–3-week intervals after heavy rainfall by core sampler for monitoring the soil moisture content during the growing season, and a gravimetric field technique was used to determine the soil moisture content in this experiment.

### 2.3 Data analysis

The data obtained was subjected to analysis of variance using R-studio-1.1.463.0 and treatment effects were compared using the Fisher's Least Significant Differences test at 5% of significance level.

## 3 Result and discussion

### 3.1 Effect of different multipurpose grass on moisture content and bulk density of the soil bund

There is no significant difference among treatments at p < 0.01 level of significance on moisture content and bulk density discussed in Table 2 both in 2020 and 2021 experimental period. However; this may not be there is no positive impact according to the authors grass biological measures with soil bund may increase the moisture content and bulk density of the soil [20]. Likewise vetiver and Sudan grass intervention have considerable impact in moisture content compare to the control (without grass stabilizer) treatment and stabilizer grasses increase the moisture content and ideal soil bulk density than constructed soil bund structures only.

**Table 2:** Moisture content and bulk density on 2020 and 2021.

Treatment	Moisture (%) (2020)	Bd (g/cm^3^) (2020)	Moisture (%) (2021)	Bd (g/cm^3^) (2021)
Desho with soil bund	6^a^	1.26^a^	8.5^a^	1.52^a^
Elephant with soil bund	6.11^a^	1.31^a^	9^a^	1.51^a^
Vetiver with soil bund	6.75^a^	1.44^a^	9.1^a^	1.44^a^
Sudan grass with soil bund	6.44^a^	1.48^a^	9.2^a^	1.36^a^
Rhodes with soil bund	5.58^a^	1.27^a^	8.4^a^	1.54^a^
Panicum with soil bund	5.6^a^	1.41^a^	8.4^a^	1.53^a^
Control	5.4^a^	1.29^a^	9.1^a^	1.56^a^
L.S.D	Ns	Ns	Ns	Ns
CV	16.42	13.24	10.98	8.87

Where; Bd = bulk density, L.S.D = list significant difference, Ns = non-significance, CV = coefficient of variation and same letters in the column indicate no significant difference. In 2021, no significant difference on moisture content and there is 13% ideal bulk density of Sudan grass with comparison of the control treatment (1.57) in the soil bund.

Moisture content and bulk density of the soil with comparison of the experiment control treatment without intervened grasses; in 2020, there was 22.2%, 17.56%, and 12.3% of percentage difference on moisture content soil in vetiver, Sudan grass, elephant and multipurpose grass respectively on soil bund structure. The bulk density of 2020 experiment period has no marked difference to prioritize among treatments which is the grass stabilizers have no impact on bulk density of the soil bund during the experiment period.

### 3.2 Effect of different multipurpose grass on adaptability and survival rate of soil bund

There was different survival rate of multipurpose grass in the experiment site to adapt and support the soil bund structure. In the trial treatments was have significant different on survival rate among the treatment of Sudan grass, Panicum and Rhodes grass have advanced survival rate which is more than 60% that are contribute biomass for fodder consumption rather than the control treatment constructing soil bund without multipurpose use grasses.

**Fig. 2:**
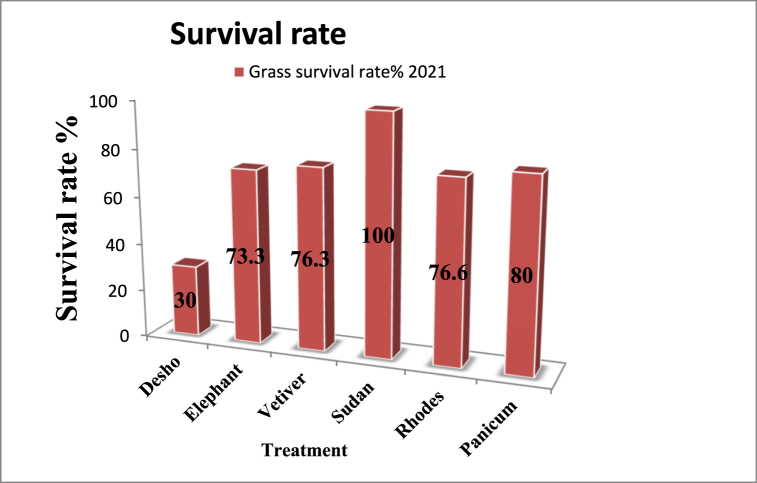
Percentage survival rate of different multipurpose grass correspondence.

**Fig. 3:**
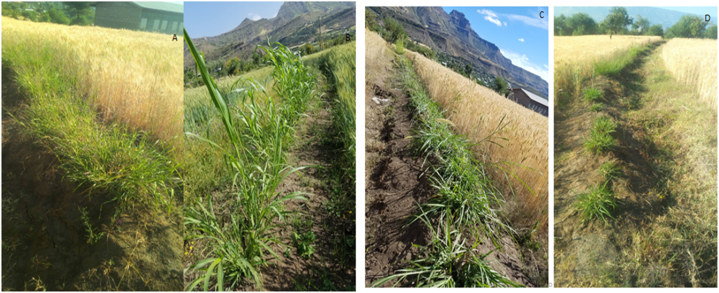
Field Performance of different multipurpose grass, where; A is panicum Grass, B is Sudan Grass, C is Elephant Grass, D is all treatment on soil bund.

Based on statistical mean square value analyses discussed in Table 3 and Fig. 2 the effect of different multipurpose grass on soil bund have highly significance difference in p < 0.01 level of significance on survival rate in 2021, whereas there is no significant difference in 2020 at (P ≤ 0.05) in survival rate (see Fig. 3).

**Table 3:** First year (2020) and second year (2021) mean square value on survival rate of trial site.

Treatment	Survival rate (2020)	Survival rate (2021)
Desho with soil bud	9.66^a^	3^c^
Elephant with soil bud	8.33^a^	7.33 ^b^
Vetiver with soil bud	9^a^	7.66 ^b^
Sudan grass with soil bud	10^a^	10^a^
Rhodes with soil bud	9.33^a^	7.66 ^b^
Panicum with soil bud	9^a^	8 ^b^
L.S.D	Ns	2.24**
CV	10	10.54

Where; l.s.d is list significant difference, Ns is non-significance, CVis coefficient of variation, ** is indicate highly significance difference and the latter is indicate difference at p is 0.05 (at 5% significance level).

### 3.3 Percentage survival rate of grass stabilizers

The higher mean survival rate is (100%) at Sudan grass (annual grass) with soil bund according to authors the grass highly drought tolerant and can give good green fodder biomass up and around to mid altitude, and the altitude of experimental site is 2326 m from average above mean see level that tells Sudan grass well perform across the area [6]. (80%) at panicum (perennial grass) with soil bund had rigorous abundance in the experiment period as [21] finding was around (70.2%) percentage of plant coverage and amazon region partaken yearly average precipitation and temperature in rainy season is 1969 ± 81 mm and 26.48 °C and in dry season 945 ± 50 mm and 27.03 °C respectively whereas the grass most probably need medium rainfall and considerable temperature can be established as the author suggested where mean maximum daily summer temperature are above 30 °C, mean daily winter temperature rarely fall below 00 °C, summer growing season rainfall ranges from 400 to 999 mm [22] and in the experiment period the rainfall, maximum and minimum temperature in 2020 were 979.2 mm, 24.5 °C and 13.5 °C respectively and in 2021 were 1027.2 mm, 24.3^0^c and 13.6^0^c, respectively, (76.6%) at Rhodes (perennial grass) with soil bund as compared the lowest survival rate (30%) of Desho grass which is effective in much more moisture area than other grasses [18] that was ideal survival rate of perennial grass range (60%) except Desho grass. In 2020 there is no significance difference among treatments at p = 0.05 level of significance and the result show that effective means of preventing sediment transport and off-site sedimentation [23].

### 3.4 Effect of different multipurpose grass on tiller, plant height and biomass in soil bund

In the trial grass have highly significance effect at p < 0.01 level of significance in tiller, plant height and biomass parameter recording but in 2020 there was no significant difference in tiller parameter at p < 0.05 level of significance in 2020 and 2021 as discussed below in Table 4.

**Table 4:** Mean square value of tiller, plant height, and biomass during the period of 2020 and 2021 at trial site.

2020				2021		
Treatment	Tiller (No)	Plant height(m)	Biomass (t/ha)	Tiller (No)	Plant height(m)	Biomass (t/ha)
Elephant with SB	26.34^a^	91.3^b^	6.7^b^	45.8^b^	85.4^b^	12.7^a^
Sudan with SB	24.60^a^	136.4^a^	20.8^a^	17.5^c^	98.7^a^	4.0^c^
Rhodes with SB	24.4^a^	69.7^c^	6.2^b^	35.4^bc^	69.5^c^	7.9^b^
Panicum with SB	27.00^a^	78.3^bc^	5.3^b^	77.2^a^	81.0^b^	10.6^ab^
LSD (5%)	Ns	16.43**	5.43**	23.8185**	8.1285**	2.945**
CV (%)	13.52	8.75	27.87	27.11	4.86	16.74

Where; Ns = non significance, No = count per single plantation, ** = highly significance difference, SB = soil bund and the same letter are not significantly different at p = 0.05 (5% level of significance).

Different grass with soil bund were had significant effect on number of tiller 27 or 77.2 (as Onyeonagu & Asiegbu, 2013 used to calculated per plantation tiller number) counts was recorded in 0.15 m^2^ area per plantation of Panicum. In 2021 which is greater tiller advantage (77.3%) as compared the lowest tiller Sudan grass, the finding is closely related with the conclusion of [24] is around 74 counts of tillers per plantation. There were no significant difference at p = 0.05 (5% level of significance) in 2020. However; Panicum (11%) percentage number of tiller difference as compared with the lowest tiller of Rhodes with soil bund treatment.

There is highly significant difference among treatments on plant height in 2020 and 2021 at p = 0.01% level of significance) accounts (98.7 cm) Sudan grass is first and followed by elephant and panicum grass 85.4 and 81 cm respectively in 2021. Whereas there is highly significant difference among treatments on plant height in 2020 at p = 0.01% level of significance) accounts (136.4 cm) Sudan grass is first and followed by Elephant and Panicum grass 91 and 78.3 cm respectively in 2020 and have slightly or no significant difference at p = 0.05% level of significance) between elephant and Panicum grass.

Effect of multipurpose grass with soil bund was highly significance at biomass (green fodder) at p = 0.01% level of significance) in 2021 was recorded (12.7 t/ha) and (10.6 t/ha) of Elephant grass and Panicum grass respectively. The biomass of Sudan grass much lower, because of the tiller population was lower that mainly affect the biomass, as scholars conclude the green forage yield significantly associated with tiller [25], related finding in Adaptation Study of Improved Elephant Grasses the highest green fodder was (37.46 t/ha) [26] with this result there is 24.76 t/ha green fodder deference because of addition of recommended fertilizer and recommended fertilizer was not used in the experiment because the experiment is applied in soil bund which is not well practiced in such structure based intervention in the study area. The value recorded in (2021) at Panicum is closely related to Ref. [21] record on Assessment of Guinea Grass Panicum coloratum under Silvopastoral Systems is (11.231 t/ha). In 2020 the result was highly significance difference in green biomass (green fodder) at p = 0.01% level of significance) the green biomass was (20.83 t/ha) at Sudan grass with soil bund and the related experiment on development of sorghum-Sudan grass hybrids for high forage yield and quality [27] average results (42.17 t/ha) and this result is half times much because of using fertilizer.

## 4 Conclusion and recommendation

Multipurpose grass have prodigious roll mainly on farm lands, range land, forest and degraded areas where conservation structure constructed for the use of additional support and or barrier for soil erosion of the structure as well as multi use to increase moisture, to have good bulk density and green fodder production.

The experiment magnify work with different grass stabilizers and soil bund have positive impact on moisture content and bulk density of the soil bund that may help full to get enough moisture for the grass and the structures to have good strength and not easily collapsed by direct runoff. The better survival rate of grass are Sudan grass, Panicum and Rhodes grass in the experiment possibly will rehabilitate moreover support the soil bund structure, blocks the direct concentrated runoff and stabilize the soil bund while the grasses will adapt in the mandate area of similar agro ecology.

The biological parameters was show significant different performance on number of tiller and plant height that affect the green forage biomass productivity of the treatments and in the experiment period Soil bund combined with Sudan grass, Elephant and Panicum Coloratum grasses 20.83 t/ha, 12.7 t/ha, 10.6 t/ha respectively have green biomass productivity advantage. Therefore; Adaptive grass with soil bund can use for green fodder production, means of additional farm land productivity trendy to implement soil and water conservation structure that may stabilize the soil bund. Result increase the productivity of physical soil conservation structure (soil bund) that will important aspect for sustainable watershed development whereas there is constraints of using biological measures for conservation, availability of other related grass and lack of technology demonstration so it is advisable to address it.

## Author contribution statement

Haymanot Lamesgn Zena: conceived and designed the experiments; analyzed and interpreted the data; contributed reagents, materials, analysis tools or data; wrote the paper.

Yalelt Abie: conceived and designed the experiments; analyzed and interpreted the data; contributed reagents, materials, analysis tools or data.

## Data availability statement

Data will be made available on request.

## Additional information

No additional information is available for this paper.

## Declaration of competing interest

The authors declare that they have no known competing financial interests or personal relationships that could have appeared to influence the work reported in this paper.
